# Osseous bridging of the condylar fossa: report of a rare anatomical variation at the outer skull base

**DOI:** 10.1007/s00276-024-03422-w

**Published:** 2024-06-26

**Authors:** Michael Wolf-Vollenbröker, Andreas Prescher

**Affiliations:** 1 Institute for Anatomy I, Medical Faculty & University Hospital Düsseldorf, Heinrich Heine University Düsseldorf, Düsseldorf, Germany; 2 Institute of Molecular and Cellular Anatomy (MOCA), Prosektur, University Hospital RWTH, Aachen, Germany

**Keywords:** Craniocervical junction, Occipital vertebra, Physical anthropology, Proatlas, Processus condylicus posterior

## Abstract

**Purpose:**

The topic of osseous variations of the craniocervical junction is a complex morphological and embryological chapter of human anatomy, with a possible impact on neurogical and vascular functionality in this morphological variable region.

**Material & methods:**

An until now undescribed anatomical variation of the exoccipital part of the occipital bone has been observed after maceration at the outer skull base of a West-European 68-year-old male body donor.

**Results:**

On both sites of the foramen magnum accessory osseous processes were observed that arise from the jugular process and point towards the lateral margin of the foramen magnum. On the left site this process forms a full arc that bridges the condylar fossa completely.

**Conclusion:**

The observed osseous bridge over the condylar fossa has not been reported on before and can be explained by the partial persistence of a primordial vertebra between atlas and occipital bone: the Proatlas. The resulting accessory structure may affect due to its topographic conditions the V3-Segment of the vertebral artery and its accompanying nerves, and thus, play a role in diagnosis and therapy of vascular and/or neurological symptoms of head and neck.

## Introduction

The transition of upper cervical spine to the occipital bone, the craniocervical junction (CCJ), of human and other vertebrates show a wide spectrum of possible accessory osseous entities [9]. The basis of this high variability are the possible different degrees of development of a primordial vertebra between atlas and occipital bone: the Proatlas [1]. A higher degree of persistence of the Proatlas leads to the formation of accessory bony structures, each of which refers to different substructures of this ancient vertebra. Even in recent years, this spectrum is widened by new findings in anatomical specimens or clinical tomographic data [11, 12, 14–16]. These new findings must always be correctly classified by the morphological sciences and examined against the background of the very extensive literature of the past 200 years. A comprehensive overview of this vast morphological chapter is given by Prescher, Menezes and Pang & Thompson [5, 7, 9].

## Materials and methods

This case of accessory osseous structures at the CCJ has been observed at the macerated outer skullbase of a 68-year-old male body donor of the body donation programme of the Medical Faculty (RWTH Aachen).

After intracervical exarticulation as the first step, the skull and upper cervical spine have been carefully skeletonized sparing the soft tissue at the CCJ. Afterwards the specimen was brought to maceration in warm water for a minimum of 72 h. This step of initial warm water maceration is followed by a stronger maceration with a solution of sodium carbonate decahydrate for several days as it has been reported by us before [14, 15]. Checking the specimens daily with focus of interest on accessory or lacking osseus or ligamentous structures at the CCJ, the maceration has been stopped and initial degreasing started by subsequent watering the skull with warm tap water. Afterwards the main step of dissolving the fatty residues has been performed with a bath in ethanol for about two weeks. After completed maceration and degreasing we examined the bony appendages of the exoccipital and supraoccipital parts of the occipital bone by using digital calipers and documented our findings photographically.

Finally, the specimen has been archived in the osteological collection of the Institute of Molecular and Cellular Anatomy (MOCA, RWTH Aachen) in Aachen.

## Results

**Fig. 1:**
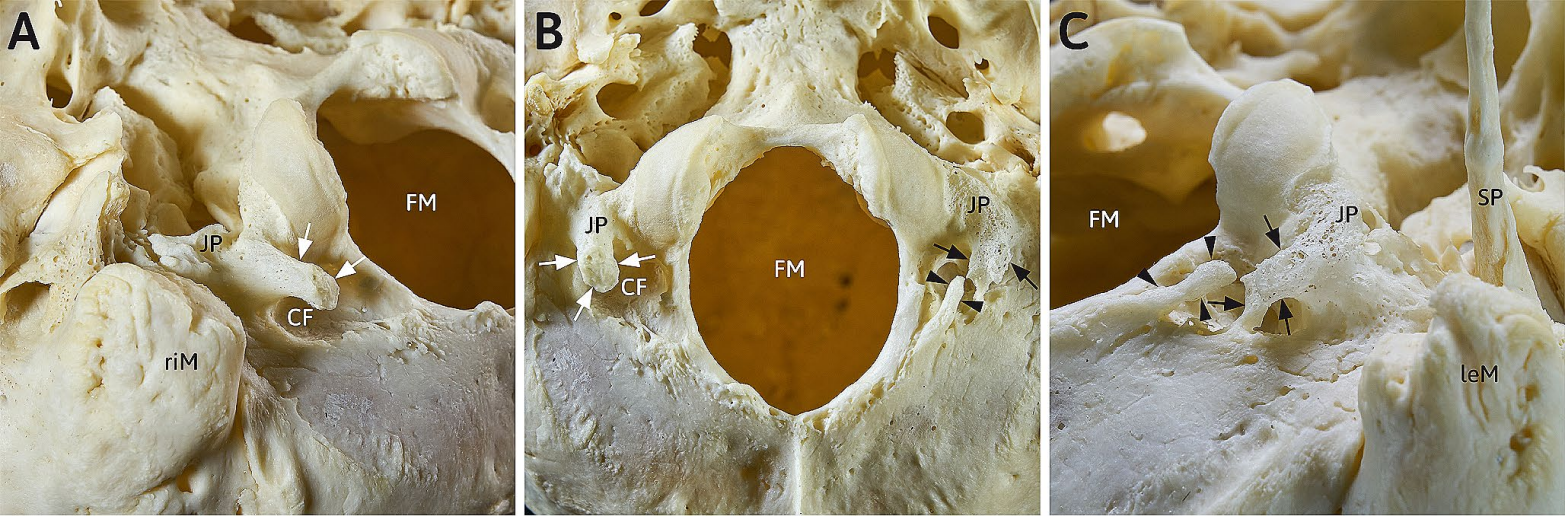
Detailed caudal view at the macerated outer skull base of a 68-year-old man, focus on region around the foramen magnum. (**A**) view from postero-latero-caudal at the right condylar fossa: a strong and sturdy bony process (white arrows) arising from the right jugular process bridges the condylar fossa (CF) und points posteromedial; (**B**) detailed median caudal view at the foramen magnum with jugular processes (JP) and arising processes an both sites; (**C**) view from postero-latero-caudal at the left condylar fossa: a bipartite bony bridge (anterior process: black arrows; posterior process: black arrowheads) spanning the condylar fossa (CF) CF: condylar fossa; FM: foramen magnum; JP: jugular process; leM: left mastoid process riM: right mastoid process; SP: styloid process

The macerated skull shows at its outer skullbase in the region of the foramen magnum two eye-catching curiosities: both jugular processes carry osseous spurs forming an arc, that is bridging the condylar fossae (Fig. 1B). Both sides differ in terms of the degree of bridging:

### Fossa condylaris dextra (figg. 1 A, 1B)

The right condylar fossa is incompletely spanned by a strong bony process. This accessory bony structure begins at the jugular process and points dorsomedially (Fig. 1B). The bony process measures 6.2 mm at its strongest portion in lateromedial dimension with a total length of 15.3 mm and does not reach the shallower area dorsal to the condylar fossa, leaving a gap of max. 4.7 mm. The process is rather sturdy and, with the exception of the dorsally pointing tip, has a rather plain surface (Fig. 1A). The roughened tip of the process lies 10.5 mm away from the lateral margin of the foramen magnum. At the bottom of the condylar fossa a condylar foramen can be observed. On examination of the outer base of the skull, no further abnormalities can be observed on the right side.

### Fossa condylaris sinistra (figg. 1B, 1 C, 2)

**Fig. 2:**
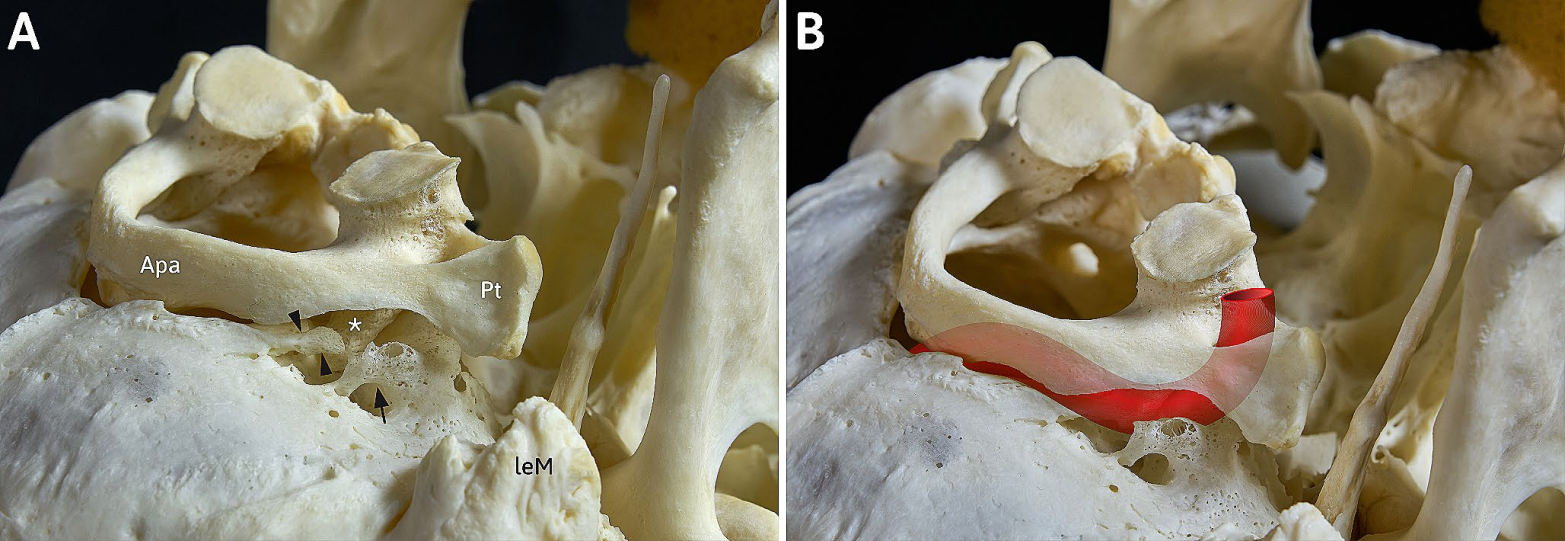
Caudal view at the macerated outer skull base and first cervical vertebra, atlas, in situ of a 68-year-old man. View from postero-latero-caudal at the osseous components of the craniocervical junction. (**A**) The examined osseous masses (black arrow and arrowheads) of the outer skull base together with the anterior spinula (*) of the atlas potentially affect the (**B**) V3-segment of the vertebral artery from cranial and are therefore comparable to other osseous variations in the same topographical position Apa: Posterior arch of atlas; leM: left mastoid process; Pt: transverse process of atlas

The left condylar fossa, on the other hand, is completely bridged by accessory bony masses. The structure is similar to the contralateral one: a bony process, rather rough and porous, arises from the jugular process and is directed dorsomedially with a total length of 18.3 mm and max. width of 5.4 mm. Unlike on the right side, this process does not end freely above the condylar fossa, but connects with the part of the occipital bone that lies further dorsal to the condylar fossa. Furthermore, another strong process (length 8.4 mm, width 2.4 mm) projects towards the first from dorsomedially. This second process (Fig. 1B, C) almost touches the first process (gap < 0.5 mm) and represents a continuation of the latter in the overview (Figs. 1B and 2A). Thus, the left condylar fossa is spanned by a bipartite bony bridge that extends from the jugular process almost to the left lateral edge of the foramen magnum. A condylar foramen can be found as well. Beside an unusual long styloid process in terms of a Processus styloideus elongatus with a length of > 47.0 mm there are no further peculiarities at the left outer skull base.

### Upper cervical spine

**Axis** – There is an inconspicuous second cervical vertebra.

**Atlas** – The first cervical vertebra shows some remarkable osseous alterations on both sites. The left and right upper articular surfaces are incompletely divided into an anterior and posterior segment with a waisting of the articular surface between both segments. Further on, accessory bony spinulae can be observed at the posterior end on the upper articular facets (Fig. 2A) and at the posterior arch at the level of where the vertebral artery loop passes on its course from the atlantic transverse processes to the posterior atlantooccipital membrane. These phenomena can be observed on both sites, but the resulting bridging of the vertebral artery is of a higher degree on the right site.

## Discussion

There are two very similar phenomena bilaterally in the area of the outer skull base at the condylar fossae. On the left side, the condylar fossa is completely bridged by an osseous arch, on the right side incompletely. These arches begin at the ipsilateral jugular process and point dorsomedial towards the posterolateral margin of the foramen magnum (Fig. 1).

The incomplete arch of the right site has to be classified as a posterior condylar process respectively Processus condylicus posterior as suggested by Schmidt & Fischer [10]. This supernumerary structure has been reported on first by Bolk in the year 1922 [2] and is, regarding the examinations of Hayek und Bystrow [3, 4], ontogenetically and phylogenetically based on the persistence of the posterior arch of the ancient Proatlas. We also assume that origin, based on own dissection and macerations studies [14, 15]. Further on, these accessory osseous processes seem to be closely related with a well-known anatomical variation of the posterior arch of the atlas, the posterior ponticle/arcuate foramen/Ponticulus atlantis posterior. This relationship comes clear due to similar topographical characteristics and may be interpreted as a common origin in the posterior Proatlas-arch [14]. Both variations consist of a bony bridge, lying cranial to the V3-segment of the vertebral artery. The posterior condylar process has its fixation point at the jugular process, the posterior ponticle is bridging the groove of the vertebral artery whereas both ends are fused to the atlas. The observed spinulae at the right site of the examined atlas form an incomplete posterior ponticle. In addition to this relationship, we developed a classification scheme of the different manifestation stages of the posterior condylar process, which comprises types I to V: in type I, there is no residual material of the posterior Proatlas-arch. In the most common type II, a partially strong ligament, the Ligamentum condylicum posterius or posterior condylar ligament, which represents a posterior part of the ligament complex of the upper cervical joint, connects the jugular process and the flat area lateral to the margin of the foramen magnum.

In type III, elongated ossicles or bony clasps are found within the ligament, but these are not in osseous continuity with the skull base. Type IV is represented by the manifestation of an anterior (IVa, posterior condylar process, Fig. 1A) or posterior (IVb), usually less strong, osseous process. The complete osseous bridging of the condylar fossa in the sense of a bony arch, as can be observed in the present case on the other, left side, represents type V (Fig. 1C), which has been hypothetical and therefore not been observed yet. This type was assumed or predicted by us in the past in the sense of an expected teratological series [14] and is now confirmed in the present specimen. Although fine bone clasps bridging the condylar fossa have been reported in the past and can certainly be observed from time to time in carefully macerated skull bases [6, 15], but in our opinion these entities do not represent the direct bony expression of the posterior Proatlas material but are merely ossifications/calcifications of the posterior condylar ligament (for details see [15]).

### Clinical significance

The clinical and practical relevance of the observed phenomena lies in the topographical conditions of these accessory osseous spurs and bridges. Lying directly cranial to the V3-segment of the vertebral artery (Fig. 2B), this may result in similar symptoms as it has been described extensively for the posterior ponticle [8], which can be found in the same topographic location. In both cases the accessory osseous structures may irritate the artery and associated sympathetic nerves, resulting in neurological (migraine cervicale, headache, neck and shoulder pain) and/or vascular symptoms [8, 15]. The surgical removement of osseous Proatlas-manifestations that affect the vertebral artery and accompanying nerves has been proposed and done successfully by v. Torklus & Gehle [13].

## Conclusion

We observed an until now not described anatomical variation of the outer skull base in the region of the condylar fossa. This structure bridges the condylar fossa and reaches from the jugular process to the area lateral to the margin of the foramen magnum. The resulting accessory bony bridge or arch is embryologically based on the partial persistence of a phylogenetical old vertebra, the Proatlas, and may lead to neurological and/or vascular symptoms. Knowledge of this variant will refine the diagnosis of such symptoms and may be the explanation for such symptoms in cases where such a phenomenon can be described in medical imaging.

## Data Availability

No datasets were generated or analysed during the current study.
